# Trends in MERS-CoV, SARS-CoV, and SARS-CoV-2 (COVID-19) Diagnosis Strategies: A Patent Review

**DOI:** 10.3389/fpubh.2020.563095

**Published:** 2020-10-27

**Authors:** José Adão Carvalho Nascimento Junior, Anamaria Mendonça Santos, Ana Maria Santos Oliveira, Adriana Gibara Guimarães, Lucindo José Quintans-Júnior, Henrique Douglas Melo Coutinho, Natália Martins, Lysandro Pinto Borges, Mairim Russo Serafini

**Affiliations:** ^1^Department of Pharmacy, Federal University of Sergipe, São Cristovão, Brazil; ^2^Posgraduate Program in Pharmaceutical Sciences, Federal University of Sergipe, São Cristovão, Brazil; ^3^Laboratory of Microbiology and Molecular Biology (LMBM), Regional University of Cariri-URCA, Crato, Brazil; ^4^Faculty of Medicine, University of Porto, Porto, Portugal; ^5^Institute for Research and Innovation in Health (i3S), University of Porto, Porto, Portugal; ^6^Laboratory of Neuropsychophysiology, Faculty of Psychology and Education Sciences, University of Porto, Porto, Portugal

**Keywords:** coronavirus (2019-nCoV), COVID-19 (condition), MERS (middle east respiratory syndrome), SARS, ELISA (enzyme linked immuno sorbent assay), isothermal amplification, RT-PCR—polymerase chain reaction with reverse transcription

## Abstract

The emergence of a new coronavirus (SARS-CoV-2) outbreak represents a challenge for the diagnostic laboratories responsible for developing test kits to identify those infected with SARS-CoV-2. Methods with rapid and accurate detection are essential to control the sources of infection, to prevent the spread of the disease and to assist decision-making by public health managers. Currently, there is a wide variety of tests available with different detection methodologies, levels of specificity and sensitivity, detection time, and with an extensive range of prices. This review therefore aimed to conduct a patent search in relation to tests for the detection of SARS-CoV, MERS-CoV, and SARS-CoV-2. The greatest number of patents identified in the search were registered between 2003 and 2011, being mainly deposited by China, the Republic of Korea, and the United States. Most of the patents used the existing RT-PCR, ELISA, and isothermal amplification methods to develop simple, sensitive, precise, easy to use, low-cost tests that reduced false-negative or false-positive results. The findings of this patent search show that an increasing number of materials and diagnostic tests for the coronavirus are being produced to identify infected individuals and combat the growth of the current pandemic; however, there is still a question in relation to the reliability of the results of these tests.

## Introduction

Coronaviruses (CoVs) are enveloped positive-sense RNA viruses that belong to the Coronaviridae family, phylogenetically subdivided into the α, β, γ, and δ genera (1). β-coronaviruses include SARS-CoV, MERS-CoV, and SARS-CoV-2 (2), with these viruses being identified as the causative agents of zoonotic infections (3). The first one, Severe Acute Respiratory Syndrome (SARS), emerged in Southern China in 2003 (1). Middle East Respiratory Syndrome (MERS-CoV) appeared in Saudi Arabia, almost a decade after the SARS-CoV outbreak (1, 3). The last one, Severe Acute Respiratory Syndrome Coronavirus 2 (SARS-CoV-2), started in the Chinese province of Guangdong in November 2019 (4).

Commercially available CoV tests currently fall into two major categories: (A) Molecular assays for detection of viral RNA using RT-PCR-based techniques or nucleic acid hybridization-related strategies and (B) Serological and immunological assays that largely rely on detecting antibodies produced by individuals as a result of exposure to the virus or on through the detection of antigenic proteins in infected individuals (5).

CoVs may cause hepatic, neuronal, and gastrointestinal diseases (6) and various symptoms, such as lower respiratory tract disease, which can lead to progressive and potentially lethal atypical pneumonia with clinical symptoms that include fever, malaise, lymphopenia, breathing difficulty, and in some cases also diarrhea (6, 7). There are many ways to transmit the virus, including close person-to-person contact, aerosol transmission, and touch transmission. “Hidden” transmission can occur through asymptomatic infected individuals transmitting the virus (8).

The rapid and accurate detection of CoVs has been shown to be useful in preventing the spread of the disease, and also in the decision-making of public health managers (9). The first step in identifying the possible presence of the virus is through taking individuals' temperatures, and physical examination may help to identify patients with a more severe condition (6). Furthermore, samples, such as saliva, nasal swabs, trachea and nasopharynx extracts, lung tissue, sputum, feces, and blood should be isolated and used for testing (10, 11). Virus isolation and viral nucleic acid detection are the principal ways of identifying the pathogen (11). Real-time reverse-transcriptase polymerase chain reaction (rRT-PCR) has been the main CoV diagnosis method, and is characterized by rapid detection, good sensitivity, and specificity (9, 12). Although PCR is the “gold standard” for virus detection, other methods have also been developed for the detection of CoVs RNA, these include several molecular, non-PCR-based methods, such as isothermal nucleic acid amplification (Loop-mediated isothermal amplification—LAMP), and nucleic acid sequence-based amplification (9).

In addition, virus detection using methods such as immunofluorescence assay, direct fluorescent antibody assay, protein microarray, semiconductor quantum dots, MAb-based rapid nucleocapsid protein detection, and microneutralization assays, that can be used to rapidly investigate the presence of viruses, have also been proposed (6, 11). Immunoassays are particularly advantageous as they can use monoclonal antibodies to detect viral antigens in <30 min without the need for expensive instruments (6).

Detection kits can accelerate accurate diagnosis, but they have different levels of test sensitivity and specificity (8, 13). The sensitivity of a test is characterized by its ability to detect a true positive, that is, to correctly identify individuals who have the disease. On the other hand, specificity identifies true negative, correctly identifying individuals who do not have the disease (14). However, achieving the specificity and sensitivity values claimed by the tests can be affected by how the tests are applied and by how the samples are treated. Parameters that can be modified include the process for the collection of the sample, its transportation and storage, and the preparation and testing of the sample (15). The tests need to be fast and reliable to identify virus outbreak sites and enable health authorities to promote appropriate measures (16).

Thus, this review aims to assess patents that address trends in strategies for the diagnosis of those infected with SARS-CoV, MERS-CoV, and SARS-CoV-2. Through providing these data, we aim to contribute to efforts to combat the current pandemic.

## Methods

**Figure 1:**
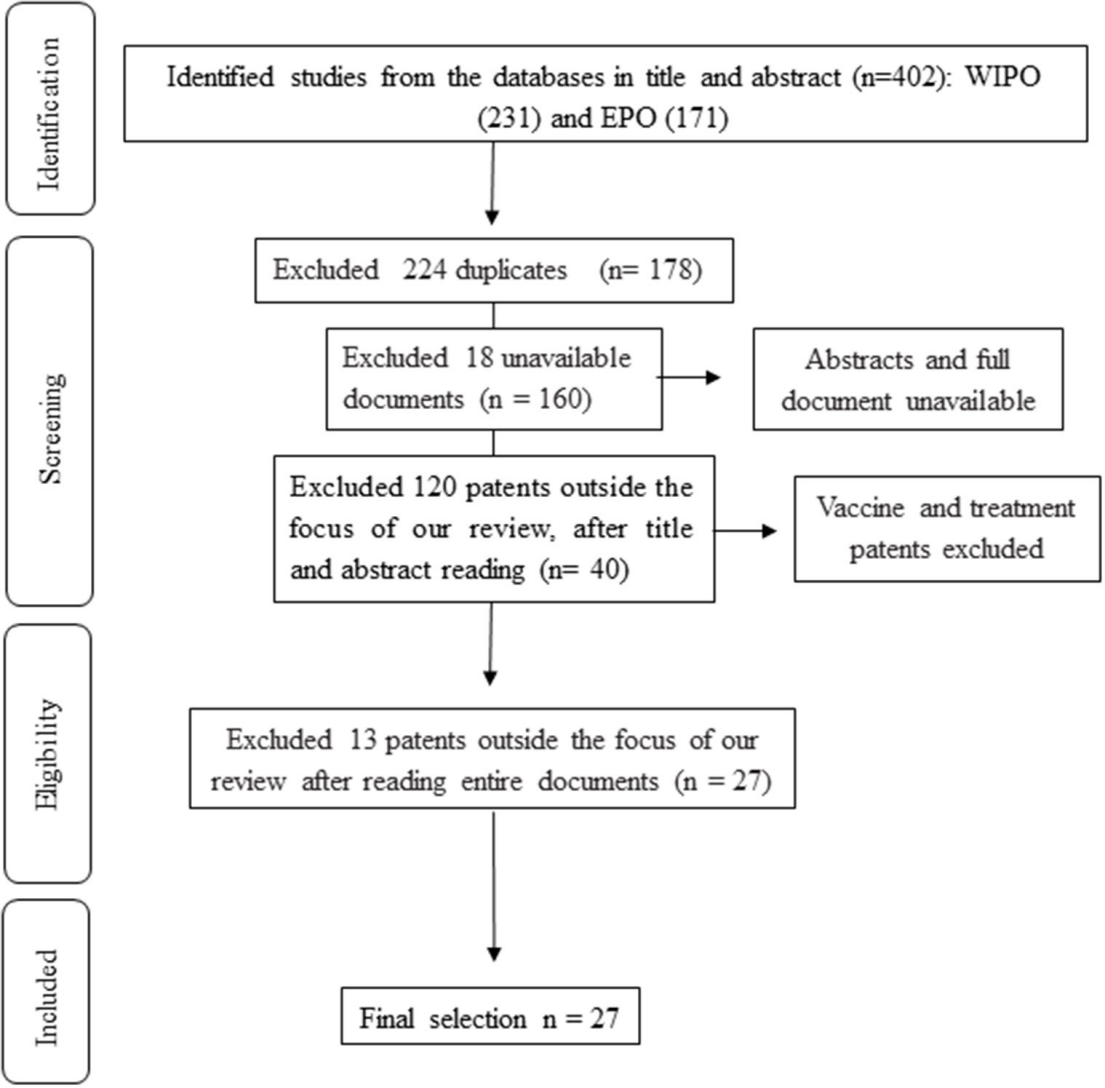
Flowchart of patent search and screening.

In the present patent review, the European Patent Office (EPO) and World Intellectual Property Organization (WIPO) databases were searched for titles and abstracts that contained the descriptors “coronavirus and MERS,” “coronavirus and COVID,” “coronavirus and 2019-nCoV,” and “coronavirus and SARS.” A total of 402 patents were identified for preliminary assessment from the databases, of which 224 were excluded due to being duplicates. After a careful check of the titles and abstracts, 120 patents were excluded for being outside the focus (diagnosis of the disease) of our review. A further 18 were excluded because the full-texts were not available. After reading the full patents 13 more patents were excluded for being outside the scope of the review. This selection process resulted in 27 patents being selected for our critical analysis according to the study objective. Figure 1 illustrates the systematic search and screening strategy used in this review, which was based on the PRISMA statement.

## Patent Search and Screening

**Figure 2:**
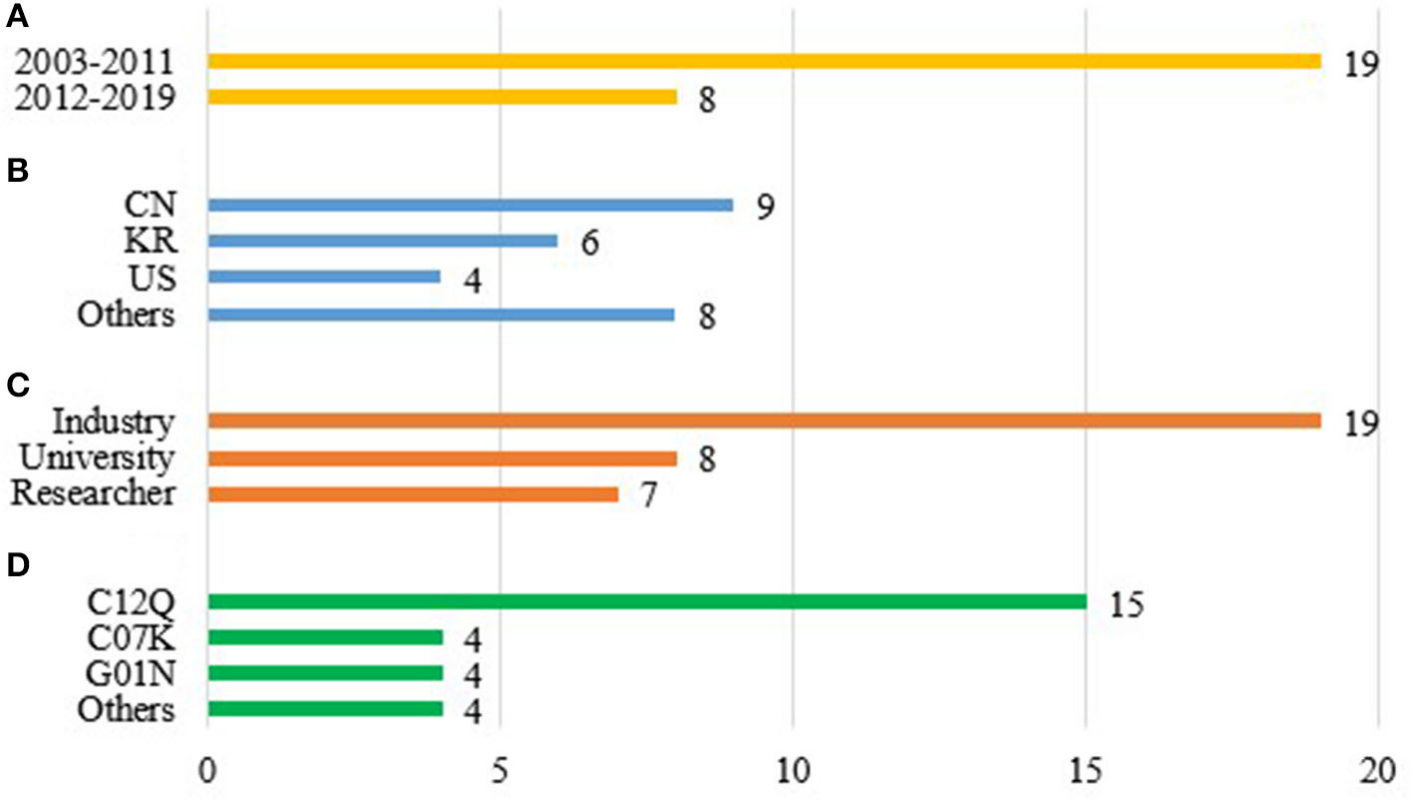
Final selection of patents. **(A)** Publication date. **(B)** Country of patent publication. CN, China; KR, Republic of Korea; US, United States of America. Others: CA, Canada; DE, Germany; JP, Japan; NL, Netherlands; SG, Singapore; WO, World Intellectual Property Organization. **(C)** Patent applicant. **(D)** International Patent Class (IPC). Others: A61K, Preparations for medical, dental, or toilet purposes; C07H, Sugars; derivatives thereof; nucleosides; nucleotides; C12N, Microorganisms or enzymes; compositions thereof; propagating, preserving, or maintaining microorganisms; mutation or genetic engineering; culture media; nucleic acids.

This review covered patents published between 2003 and 2020, a period that encompasses the emergence of SARS-CoV epidemics in Asia, MERS-CoV in Saudi Arabia, and the current SARS-CoV-2 pandemic. The largest number of patents on diagnostic methods targeting these pathogens were registered between 2003 and 2011 (19 patents) (Figure 2A).

China (CN) produced the most patents, with nine, followed by the Republic of Korea (KR) with six patents, and the United States (US) with four patents (Figure 2B). These countries are usually well-represented in the field of patent filing due to their advanced technological and scientific sectors and, in this area, their strong records of innovation in the production of disease diagnosis methods. Moreover, a large number of healthcare and biotechnology companies (including startups) are based in these countries which are world leaders in the sector of diagnostics and molecular biology (17, 18).

A patent can be applied for by several different scientific entities, including industrial laboratories, universities, and/or independent researchers. As expected, the industrial laboratory sector applied for the largest number of patents (19 out of a total of 27 patent applications) (Figure 2C). In the industrial sector, companies applying for patents included Gen-Probe Incorporated, Biomerieux B.V., Beijing Applied Biological Tech Co. Ltd., Adaltis Inc., the Korea Research Institute of Bioscience and Biotechnology, Shanghai Institute Biological Sciences, Mogam Biotechnology Institute, and Samsung Electronics Co. Ltd. Universities filed eight patents, sometimes in partnership with companies, and sometimes independently. Of the patents identified in the review, seven were filed by independendent researchers. The cooperative process between universities and industry for the development of innovative health products and in other areas is a global trend that yields benefits for both universities and industry, and, ultimately, for society (19). Every patent filed has an International Patent Classification (IPC), which classifies the invention based on the technological area to which it belongs. In the present review, the code C12Q, which refers to “*Measuring or testing processes involving enzymes, nucleic acids or microorganisms; compositions or test papers therefore, processes of preparing such compositions; condition-responsive control in microbiological or enzymological processes”* was presented in 15 patents (Figure 2D), followed by C07K (Peptides) and G01N (Investigating or analyzing materials by determining their chemical or physical properties) with four patents each. The patents identified in the review used two main techniques target amplification and enzyme linked immunosorbent assay (ELISA).

## Techniques and Methods Used in the Patents

### Target Amplification Techniques

Amplification techniques seek to use different methods to repeatedly amplify certain regions of a genetic material present in the sample to detectable levels of diagnostic. Different methods improve both the sensitivity and specificity of technique, whether by adding oligonucleotides and enzymes or by controlling specific reaction conditions. Most methods are automated and provide quantitative and accurate results in a short period of time. They also eliminate the need for specialized training and reduce the risk of contamination and human error (20). These techniques can produce cost-effective, reproducible tests with high sensitivity and specificity that provide reliable diagnoses (21). There are two main amplification techniques: polymerase chain reaction (PCR), and isothermal amplification technologies (IAT), each with a number of methods that are described below.

#### Polymerase Chain Reaction (PCR)

PCR is an enzymatic method that separates the two strands of DNA to produce numerous copies of a gene, using a primer to mark the location and a DNA polymerase to continuously assemble a copy in each segment (9). Real time reverse-transcriptase PCR (rRT-PCR) is a recently developed PCR-based detection method used to detect and quantify multiple species from a sample (22). Viral antigens, viral RNA, DNA, and biomarkers can be detected using rRT-PCR blood/serum and tissue samples (23). rRT-PCR is a popular method as it has multiple advantages including its speed in providing a simple and sensitive quantitative assay (9, 24). However, there have been situations in which rRT-PCR has produced false positives, thereby limiting its clinical use in detection (25). Another recognized disadvantage of rRT-PCR is its relative high costs related to equipment acquisition, maintenance and the required reagents when compared to other methodologies (26). It is noteworthy that a lack of the reagents required for rRT-PCR during the SARS-CoV-2 outbreak has been a serious limitation on the use of this methodology, especially in developing countries (27).

The amplified product and probe melting during rRT-PCR were recognized by continuous fluorescence (25), and is measured after each cycle, with its intensity reflecting the amount of DNA in the sample at a time-specific (28). Several kinds of rRT-PCR have been developed such as multiplex rRT-PCR, which produces results based on the amplicon size of the pathogen in gel electrophoresis. However, this method exhibits some disadvantages in comparison to conventional PCR, such as the inability to monitor the amplicon size without opening the system and its incompatibility with some other platforms (29). Quantitative rRT-PCR (RT-qPCR) uses the same methodology, but the technique is more efficient than multiplex rRT-PCR, and also avoids contamination (30).

RT-PCR test kits suffer from some limitations, such as their complicated operation; long turnaround times, taking on average over 2–3 h to generate the results; their inability to function with a low viral load or with samples that have not been very carefully collected; variation in the diagnosis rate; and the requirement of expensive equipment and trained technicians to use them (31, 32). The experimental method leads to the efficiency of the technique. For example, in RT-PCR tests the number of thermal cycles can reflect in the concentration of viral RNA, through the increase of the cycles leading to higher uncertainty in the test accuracy, due to the small viral load (33). For this reason, high false-negative rates of COVID-19 infection have been reported (34).

##### Patents identified in the review using PCR

Table 1 presents a summary of published patents of SARS and MERS coronavirus tests. Mei et al. (39), in 2004, patented a multi-channel combined micro-fluidic chip suitable for SARS virus detection. The invention uses the PCR method to realize RT-PCR intermodal detection to provide better accuracy, sensitivity, stability, ease of operation, and fast detection. The test takes 25 min and is low-cost because the primer does not need a fluorescent label. In general, nested double PCR is used to detect pathogens; however, its operation is considered tedious and accuracy is only 40–60%. However, SARS virus can be detected at very low concentrations, with the detection limit reaching 10^−2^ copies/100 μl. The type of sample used is the patient's saliva. The microfluidic chip is based on a pipeline, a liquid containing tank, and a reaction tank arranged on a sample feeding pipeline between the liquid containing tank. The materials that compose the PCR micro-fluidic chip such as polycarbonate (PC) plastic, quartz, and glass are resistant to light permeability. The structure of the chip comprises a plurality of liquid reservoirs, microchannels, and millimeter-sized reaction tubes or reaction wells (pools) (62, 63).

**Table 1:** Published patents of SARS and MERS coronavirus tests.

**References**	**Year**	**Country**	**Product**	**Test type**	**Target**	**Sample type and/or result time**	**Benefits**
Wang (35)	2003	CN	Reagent kit diagnosis of SARS-CoV antibody	IBT	IgG	Serum sample	Comprehensive antigens, high sensitivity and strong specificity
Briese et al. (36)	2004	US	Synthetic nucleic acid sequence to detect SARS-CoV	RT-PCR	N gene	Feces and blood.	High sensitivity and specificity
Che et al. (37)	2004	CN	Monoclonal antibodies that bind to the SARS-CoV N protein	ELISA	IgG1 or IgG2b	Serum and lung tissue	High specificity, good repeatability, easy operation, low cost
Houde and Lacroix (38)	2004	CA	Diagnostic peptides for SARS-CoV	ELISA, Immunochromatography; Antigen filter test	IgG	Serum or biological fluid	High sensitivity and specificity
Mei et al. (39)	2004	CN	Multi-channel combined microfluidic chip to detect SARS-CoV	rRT-PCR	NS	Saliva. 25 min.	High sensitivity, precise, stable and easy to operate, specificity, fast detection speed, low cost
Artus Ges Fuer Molekular Biolog (40)	2004	DE	SARS-CoV detection kit	rrRT-PCR	NS	Sputum, feces, or blood.	Efficient, sensitive and reliable
Sillekens and Biomerieux (41)	2004	NL	Nucleic acid sequences as primers for detection of SARS-CoV	NASBA	N gene	Nasopharyngeal aspiration, feces, or blood	NS
Vijaysri et al. (42)	2004	CA	Oligonucleotide for detecting SARS-CoV	Amplification test	Rep gene	Hours.	Sensitivity
Wu and Gao (43)	2004	CN	Short-handled circular probe system	rRT-PCR	NS	Blood. 2 h.	High specificity and sensitivity
Inoue and Hong (44)	2005	SG	Test to detect SARS-CoV	rRT-PCR	NSP1	Plasma, throat swab, sérum, saliva, sputum. Hours.	Fast, sensitive and specific compared to available commercial tests
Kostrikis (45)	2005	US	Multi-allelic molecular detection of SARS-CoV	rRT-PCR	S, E, M and N genes	Nasopharyngeal aspiration, feces, or whole blood	High sensitivity and specificity
Lim et al. (46)	2005	KR	Primer set for detecting SARS-CoV silicone-based micro PCR chip	rRT-PCR	ORF1ab, S, E, M, N genes	30 min.	Reduces the possibility of determining false or false positives; detect SARS virus without cross-reactivity
Ma and Jie (47)	2005	CN	Antibody against a SARS-CoV NC protein	Test strip	N protein	Serum, plasma, urine, semen, saliva, sweat, tears. 10 min.	Sensitivity 10 pg/ml recombinant protein N
Minekawa et al. (48)	2005	JP	SARS-CoV detection method	RT-LAMP	ORF1ab, R2, R3	Any sample derived from human living body; 20–35 min.	High sensitivity and speed, does not require temperature control
Qin et al. (49)	2005	CN	Antigenic determinant of SARS-CoV NC protein epitope	Enzyme immunoassay	IgG and IgM	Sputum or serum	High affinity for SARS anti-virus antibodies, simple, sensitive method and high precision
Park et al. (50)	2006	KR	Oligonucleotides to detect SARS	rRT-PCR	NS	Feces	Detects virus at the initial stage. Good specificity and sensitivity
Park (51)	2008	KR	Detection of SARS by NC antigen or S protein	ELISA or PCR	IgG or N and S gene	Blood	Fast and safe
Lou et al. (52)	2009	US	Oligonucleotide for detecting SARS-CoV	All types of amplification reactions	Rep gene	Any sample that contains SARS nucleic acid. Hours.	Analytical sensitivity and specificity
Kacian (53)	2010	US	Detection probe for SARS-CoV	TMA	ORF1ab genes	Nasopharyngeal swab	Selective and sensitive detection
Jeong et al. (54)	2012	KR	Aptamer specific to SARS-CoV	rRT-PCR or ELISA	N protein or IgG	NS	NS
Kaiyuan et al. (55)	2012	CN	Multiplex fluorescent PCR in tube for 5 types of CoV—OC43, 229E, NL63, HKU1 and SARS	rRT-PCR	NS	Nasopharyngeal swab	Detection of 5 types of CoV in one tube. Sensitive, fast, accurate, saves materials, and reagents
Yana et al. (56)	2017	JP	Antibodies against MERS-NP	ELISA, Immunochromatography; Antigen filter test	IgG	Serum, plasma, urine, semen, saliva, sweat, tears.	Specifically detects only MERS-CoV. Precision, speed and simplicity.
Ahn et al. (57)	2018	KR	Primer set for detection MERS-CoV	RT-LAMP	ORF1b and N gene	Sputum, lung tissue	High specificity, does not need temperature control, or expensive equipment
Wang et al. (58)	2018	CN	Primer probe set and kit for detecting SARS-CoV and MERS-CoV	RPA	NS	25 min.	Short time, good specificity, minimum detection limit, lower cost, prevention of false negatives
Zhou et al. (59)	2018	CN	Fluorescent primer MERS-CoV	rRT-RAA	NS	Throat Swab; 20 min.	Closed reaction, does not depend on PCR, tested at normal temperature 37–39°C. High specificity and sensitivity
Han et al. (60)	2019	KR	Fusion protein based on MERS-CoV NC and mAbs	ELISA	IgG	Blood, body fluid, saliva, and sputum.	Standard positive/negative control; High sensitivity and specificity.
Jeong et al. (61)	2019	WO/KR	Antibody to detect MERS-CoV binding to the fusion protein of the N-terminal and C-terminal domain fragment of the NC protein	ELISA	IgG	10–15 min	High specificity

ITB, Immunoblotting; mAbs, Monoclonal antibodies; NASBA, Nucleic acid sequence-based amplification; NS, Not Specified; NSP1, Non-structural protein 1; RPA, Recombinase Polymerase Amplification; RT-LAMP, Reverse Transcription Loop-Mediated Isothermal Amplification Method; rRT-PCR, Real time Reverse Transcription Polymerase Chain Reaction; RT-PCR, Reverse Transcription Polymerase Chain Reaction; rRT-RAA, Real Time Reverse Transcription Recombinase Aid Amplification; RT-RAA, Reverse Transcription Recombinase Aid Amplification; TMA, Transcription-Mediated Amplification; WO, World Intellectual Property Organization.

*Countries: CN, China; JP, Japan; KR, Republic of Korea; TW, Taiwan; US, United States of America*.

Artus Ges Fuer Molekular Biolog (40) in 2004 described a quantitative real-time PCR method for SARS detection. The kits use oligonucleotides to detect the SARS virus using a biological sample, such as body fluid (in particular sputum), feces or blood. The inventors state the invention provides an efficient, sensitive, and reliable qRT-PCR method for virus detection, and is able to detect all known variants of the virus and excluded all other near-related Coronaviridae. The method brings as an advantage, the quantitative detection of SARS-associated virus with a theoretical yield point of 10 genome, corresponding to 120 RNA copies per ml of the biological sample. Therefore, the RT-PCR method is correct in at least 95% of the cases examined.

Briese et al. (36) in 2004, developed a PCR and RT-PCR assay for SARS-CoV detection. The invention uses biological samples, such as body fluids, including cerebrospinal fluid, pericardial fluid, peritoneal fluid, saliva, serum, feces, and urine, and allows a rapid, sensitive and specific molecular diagnosis of the virus. In addition, the invention provides a synthetic nucleic acid sequence comprising 10–30 consecutive nucleotides, including the N region of SARS. Moreover, the synthesized DNA strands can subsequently serve as additional templates for the same primer sequences, and the PCR can therefore be used to detect the existence of a defined sequence in a DNA sample. The sensitivity of the method showed 500 copies in ~100 ng of total RNA extracted post-mortem from a SARS victim.

Wu and Gao (43) in 2004 patented a target sequence to identify SARS-related CoV using a gene diagnosis technology with an RT-PCR primer, a short-handle circular ring probe, and a kit. The invention uses blood to determine the presence of the virus, in a rapidly, timely, specific and sensitive way. The detection can be done in 2 h, for 1–10 numbers of copies, which greatly improves the sensitivity of hybridization detection. Moreover, the target point selection was performed with a stable point without a mutated region having a length in the range of 120–180 bp. The invention kit includes the RNA extract, the PCR solution, a positive control, and a self-contained reagent.

The invention by Inoue and Hong (44), in 2005, provided a simple, sensitive, and specific diagnostic test when compared to three commercial tests available. A one-step PCR method rather than the usual two was used to detect SARS-CoV. This test is based on a qualitative nucleic acid amplification assay for the detection of SARS-CoV in patient samples, such as plasma, throat swab, serum, saliva, sputum, and uses specific primer pairs designed from the SARS-CoV non-structural protein 1 (NSPl), a putative proteinase. The invention provides a gel-based RT-PCR detection kit, which includes one or more primers and/or probes, and may contain a positive control nucleic acid or at least a portion, thereof comprising the NSPl region, as either RNA or DNA.

The primers used in the invention should be between 16 and 20 nucleotides in length, and the amplification product can be detected by determining the amplification product' length. The preferred part of the NSP1 region for amplification is between 4,609 and 7,003 nucleotides. Three other tests (Eiken, Artus, and Roche SARS diagnostics) were used to compare the efficiency of the invention, which provided the most sensitive detection. The invention is sufficiently sensitive to detect a few molecules of RNA in each RT-PCR reaction, with the results being acquired in hours.

Kostrikis (45) in 2005, described a molecular-beacon-based multi-allelic qRT-PCR assay for the detection and discrimination between SARS-associated and other CoV isolates in clinical samples, such as nasopharyngeal aspirate, stool or whole blood. The method comprises mismatch-tolerant molecular beacons, four sets of PCR primers for four different viral genes, and four different molecular beacons, an exogenous RNA standard that is added to the sample that can be reverse-transcribed and amplified by one of the primer sets, and a fifth molecular beacon that is labeled with a different fluorophore, specific for the exogenous RNA standard.

The multiple targets sequences of the invention are the S, E, M, and N genes in the SARS-CoV. The samples tested using the four genes showed 100% specificity. Therefore, the detection of the four target alleles in the same tube minimizes the likelihood of missing the presence of the virus in the sample, and increases the sensitivity and specificity of the method. The kit contains reagents for performing amplification reactions including PCR and also for sample pretreatment including the reagent required for CoV release and/or purification (45).

In 2005, Lim et al. (46) developed a PCR method and kit for detecting SARS-CoV. This invention provides a set of primer-CoV specific primers, and the targets were the ORF1ab, S, E, M, N genes of the virus. The primer set could specifically detect a virus without cross-reactivity with other CoV, and reducing the possibility of detecting false or false positives. PCR can be carried out in a variety of materials, such as polypropylene tubes, a 96-well plate, or in a silicon-based micro PCR chip. However, when used in a silicon-based micro PCR chip, PCR also can be carried out by thermal cycling, shortening the reaction to 30 min.

Park et al. (50) in 2006, patented a kit to detect SARS using oligonucleotides including a primer and/or a probe, designed to be more sensitive and specific than conventional tests. The invention could detect the early stage of infection using RT-PCR and biological samples such as feces to isolate and purify viral RNA. The method can mix the enzymes comprising the DNA polymerase and/or reverse transcriptase with a reaction mixture comprising the oligonucleotides, and can add an RNA specimen to the prepared mixture, and the amplifying reaction solution comprising the specimen RNA prepared using an RT-PCR process.

In 2008, Park (51) described a method using nucleocapsid or a spike protein antigen to diagnose SARS-CoV. The invention includes a solution containing SARS ATP-ceramide-N_monoclonal antibody, a chromogenic substance. The antibody comprises a monoclonal antibody, and the chromophore comprises an enzyme or gold. The method uses blood as a sample type, and IgG or N and S genes as a target to detect the virus. The patient can only be diagnosed if two different samples test positive or the same sample tests positive twice.

In 2012, Jeong et al. (54) formulated an oligonucleotide of a specific sequence and pharmaceutical composition as a physiologically acceptable carrier that can be used for detecting SARS-CoV. The invention provides a method of treatment and diagnosis using an oligonucleotide aptamer that has a special affinity with the nucleocapsid, and has more effect than an antibody, being smaller in size. Furthermore, the nucleotide molecule is not sensitive to temperature changes, and regenerates within a short time. The target used in this invention can be an N protein or IgG. The aptamers used in the invention include single-stranded DNA ligands because they have a high affinity and complex structure that binds to the target protein and can be identified using the systemic evolution of ligands by exponential enrichment (SELEX). The SELEX method separates high-affinity DNA and RNA ligands into the target molecules, including proteins and organic small molecules. The pharmaceutical composition may be prepared by mixing and using substances, such as lubricants, disintegrants, solubilizers, dispersants, stabilizers, suspending agents, pigments, and others. However, its use is limited as samples analyzed by this method need to be processed in certified laboratories, causing a delay in the results (64).

In 2012, Kaiyuan et al. (55) described a tube multiplex fluorescent PCR detection method for five types of human coronavirus, OC43, 229E, NL63, HKU1, and SARS, which can be used as a detection reagent for scientific research and clinical uses. The sample used in this technique is a nasopharyngeal swab, and the probes can hybridize with the nucleic acid sequence amplified by the primers.

#### Isothermal Amplification of the Target

Unlike PCR, isothermal amplification methods require only one temperature, thus eliminating the need for thermal cyclers (65). The method is fast, sensitive, does not require strong energy sources, and is easy to implement in service locations or in situations with limited resources. However, the method has some disadvantages, such as the challenge of designing compatible primer pairs, the potential to generate non-specific amplified products (20, 66). Target isothermal amplification technologies include nucleic acid sequence-based amplification (NASBA), transcription-mediated amplification (TMA), loop-mediated isothermal amplification (LAMP), recombinases aided amplification (RAA), and recombinase polymerase amplification (RPA), which are discussed below (67).

Introduced in 1991 by Compton, NASBA is a technique commonly used for selective amplification of RNA fragments (68). It is a transcription-sensitive system, ideal for specific replication of nucleic acids *in vitro* (69), which uses two specific oligonucleotide primers and three avian myeloblastosis virus (AMV) reverse transcriptase enzymes (70). RNAase H, RNA polymerase, and T7, together with the primers, amplify the RNA targets at 41°C (71), producing at the end of the reaction the terminal product, ssRNA, detected by methods, such as electrochemiluminescence, gel electrophoresis, sphere-based enzymatic detection, and enzyme-linked gel assay (20).

Similar to NASBA, TMA also uses isothermal amplification with the use of reverse transcriptase to produce cDNA from the target RNA. RNA polymerase then generates complementary RNA derived from cDNA, thus amplifying the original target RNA of interest. It presents fast kinetics, producing up to 1,000 copies of target RNA per reaction. The obtained amplicons can be detected by gel electrophoresis or oligonucleotide probes (20, 72). Like NASBA, it needs the reaction temperature needs to be carefully controlled to denature the secondary structures. The results from commercially available TMA assays for Human Immunodeficiency Virus (HIV), Hepatitis C Virus (HCV), and Hepatitis B Virus (HBV) are similar to those for commercial RT-PCR (73).

Over the past 10 years, LAMP has become a frequently used technique due to its effectiveness, sensitivity, and specificity for diagnosis. The method is based on the use of four specific external and internal primers responsible for amplifying nucleic acid (65). It presents fast detection (around an hour), ease-of-use, and only a single temperature for incubation (74). Several LAMP assays have been applied to detect a variety of pathogens, such as parasites, bacteria, and viruses, including influenza, Ebola, Zika, yellow fever, MERS-CoV, and SARS-CoV-2 (75). Moreover, it can be performed with a variety of samples, such as blood, urine, saliva, and semen (76).

In a study by Wang et al. (77), the LAMP assay demonstrated 100% sensitivity and specificity, with the reaction being completed in 60 min, while a RT-PCR assay required 82 min. The results obtained were visual and easy to observe. The method appeared to be a powerful tool to monitor suspected patients and risk groups through the identification of SARS-CoV-2. A study by Park et al. (78) used a non-purified sample directly with the LAMP technique because its high amplification efficiency made it possible to detect the results through colorimetric methods. Studies have already demonstrated that the RT-LAMP assay can be used for MERS-CoV detection, using primers directed to the viral N protein sequence.

RPA is characterized by a minimum need for sample preparation, a low operating temperature (37°C), the use of freeze-dried reagents, simplicity, sensitivity, selectivity, and rapid amplification (about 104 times in 10 min). This technique uses two primers and one probe, and the unwinding of the DNA and annealing of the primers uses recombinase enzymes (79, 80). It can use several samples, such as blood, serum, plasma, feces, urine. Also, as it is reagents have been freeze dried, the RPA kit can be kept at room temperature for several months (81).

RAA makes use of two primers, three specific enzymes, and three proteins to amplify DNA at 39°C in about 30 min. The enzymes include a UvsX recombinase extracted from *E. coli* to anneal the model DNA primers, single-stranded DNA binding protein (SSB), to form a D-loop structure to maintain a single-stranded state of model DNA, with the help of DNA polymerase for amplification and extension (82). In this methods, it is possible to use reverse transcriptase with or without a fluorescent probe for real-time detection of RNA amplicons (83, 84) has high specificity and sensitivity, is easy to use, and can produce a clinical diagnosis in minutes (85).

##### Patents using isothermal amplification of the target

In the patents identified in this review, Silleke and Biomerieux B.V. (41) used nucleic acid sequences as primers for SARS-CoV detection through NASBA. The target regions chosen for amplification correspond to the gene that encodes the SARS-CoV nucleocapsid protein. The invention also addresses the use of the methodology and the proposed primers to quantify the virus before and after therapy, through sample collection from nasopharyngeal aspiration, feces, or blood. When the analytical sensitivity of the primers was evaluated, they showed 2.5 copies of RNA *in vitro* in the amplification (41).

The 2005 invention by Mineka et al. (48) also uses a method to detect SARS-CoV through RT-LAMP by detecting ORF1ab, R2, and R3 genes in 20 to 35 min, with the presence of 2.5–10 copies. An oligonucleotide primer was first prepared that selectively hybridizes to a nucleotide sequence specific to the SARS-CoV, and then uses the LAMP method to identify the virus.

The 2010 patent by Kacian and Gen-Probe Inc. (53), describes a detection method for SARS-CoV through TMA. The tests obtained a sensitivity of 100–1,000 copies, having 100% reactivity, with an endpoint of detection of 80 copies/mL. The tests were performed using a nasopharyngeal swab as a sample. In assessing specificity and sensitivity, the detection probe did not cross-react with HIV, HBV, parvovirus, and HCoV-229E (viral nucleic acid from the human coronavirus strain).

The 2018 patent by Ahn et al. (57) used primers targeting the ORF1b and N genes in sputum and lung tissue samples to identify MERS-CoV. The method has high specificity and does not require temperature control so the equipment was relatively inexpensive. To verify the specificity of the primers in relation to MERS-CoV six different viruses were used: influenza-A (H1), influenza-A (H3), influenza-B1, influenza-B2, human metapneumovirus (MPV), and 229E were submitted to RT-LAMP. The primers amplified only the sample containing MERS-CoV, thus proving its specificity. Finally, the efficiency of LAMP was compared with that of RT-PCR assays available in the market, and was shown to have better sensitivity and a shorter reaction time.

The 2018 patent by Wang et al. (58) describes a kit to detect SARS-CoV and MERS-CoV using RPA. The specificity of the probe and primer were evaluated and no cross-reactions with other types of viruses tested were observed. The minimum detection limit was 10 copies in the SARS-CoV model and 100 copies in the MERS-CoV model. The tests were shown to have a useful life 1 year, being one of the main advantages of this test, in addition to its low-cost and prevention of false negatives.

The patent by Zhou et al. (59) published in 2018 describes a reverse transcription RAA (RT-RAA) method for detecting MERS-CoV using a primer and a fluorescent probe using. The tests take ~20 min and use a nasopharyngeal swab sample. It presented a detection limit of 10 copies/mL and specificity was proven by submitting common pathogens, such as influenza-A H1N1 virus, influenza-N, respiratory syncytial virus and rhinovirus to the method.

### Enzyme Linked Immunosorbent Assay (ELISA)

Finally, quantitative analytical methods that perform antigen-antibody reactions through colorimetric change with the aid of an enzyme conjugate and substrate are useful for quantitative and qualitative results in respect of molecules in biological fluids. One of the most popular of these methods is the enzyme linked immunosorbent assay (ELISA) because it can be used to quantify substances at very-low concentrations (86).

This method consists of an analytical biochemical assay with high sensitivity and specificity for the detection and qualitative or quantitative analysis of an analyte without using expensive and sophisticated devices. Any substance, whether it is a specific protein or a mixture of it, can be used as an analyte. Its methodology comprises the production of monoclonal or polyclonal antibodies using antigens. Radioimmunoassay techniques, with the use of radioisotopes or fluorescence markers, are often selected to detect proteins. In the latter method, protein quantification occurs indirectly, with the absorbance of the color generated by the chemical bond due to the the presence of the dye being proportional to the amount of protein. These techniques demonstrate good sensitivity and detection limits, and ability to quantify below the nanoscale (87). A study developed by Xiang et al. (88) reported the use of ELISA in tests for IgM and IgG antibodies directed at the diagnosis of COVID-19, obtaining strong sensitivity and specificity in relation to their detection.

However, false-negative results can occur in tests based on antibody detection. The IgM antibodies are produced as part of the early immune response during the initial stage of the infection, while the IgG antibodies indicate that the disease has entered a recovery period, or may be present if there has been prior infection (89, 90). The antibody tests are used in cases where RT-PCR is negative and there is an epidemiological bond to SARS-CoV-2 infection and during the period when symptons are first presented and the viral load is high (91). The false-negative cases, in this type of test, can occur in situations where the antibodies are close to the germline, being able to bind to the SARS- CoV-2 antigens (92). Another issue that the immunological assay presents is the high incidence of false-positive cases in seronegative patients. This is probably related to inappropriate sample collection time in relation to the stage of the infection, as well as to naive IgM antibodies, which can produce an incorrect result due to the antibodies low action. However, the search for class-switched isotypes, in this case IgG, might help to decrease the risk of errors. In addition, a target antigen is also essential when the virus being tested is capable of mutating because the same viral antigens will be present in its structure (93).

#### Patents Using ELISA

In 2004, Che et al. (37) developed and patented a group of monoclonal antibodies belonging to IgG1 or IgG2b with specific binding capacity to the SARS-CoV N protein through a hybridoma, in addition to providing the reagent for SARS-CoV antigen deletion. In a clinical application study, the double antibody sandwich ELISA kit detected the SARS-CoV antigen in the patients' serum, obtaining high specificity and sensitivity. There was no cross-reactivity with cell cultures that were not infected with SARS-CoV.

Houde and Lacroix (38) in 2004, created an *in vitro* diagnostic method for detecting the presence or absence of antibodies indicative of SARS-CoV by binding them to a peptide, or analog of it, to form an immune complex. In the ELISA assay the peptide was adsorbed or covalently coupled in wells of a microtiter plate treated with the serum, or the biological fluid to be tested. After washing with anti-human IgG or anti-human IgM, IgA was labeled with peroxidase and added to the wells and for peroxidase determination with a corresponding substrate. Clinical samples can range from cultured cells, cell supernatants, cell lysates, serum, plasma, biological fluid to tissue samples.

In 2005, Qin et al. (49) developed the SARS-CoV nucleocapsid protein epitope. This polypeptide has a high affinity for SARS anti-virus antibodies and the antibody developed in the invention has a high affinity for SARS-CoV. The detection method was reported to be simple, sensitive and with high precision. It uses IgG and IgM and serum or sputum samples from patients to detect the virus. Park (51) formulated a method to diagnose SARS-CoV using a nucleocapsid protein antigen (SARS-CoV-N) or spike protein (SARS-CoV-S). The method uses HRP-conjugated human anti-IgG antibody or the SARS-CoV-N monoclonal antibody mixed with a sample containing SARS-CoV to adsorb the SARS-CoV-N antibody. Finally, in 2019, Jeong et al. (61) and Han et al. (60) developed an antigen for MERS-CoV diagnosis using the NC fusion protein which included a fragment of the N-terminal domain and a fragment of the C-terminal domain of the CoV N protein. They used IgG and compared their innovations with a kit already available in the market from Euroimmun®. The invention by Han et al. showed positive results for human serum diluted 128 times, while the commercial kit showed positive results only in serum diluted 16 times. This patent used blocking ELISA, and as samples blood, body fluid, saliva, and sputum. Although both patents revealed high specificity, the latter one exhibited higher sensitivity (60).

## Diagnostic Tests for COVID-19

A similar search of Google Patents was performed using the keywords “SARS-CoV-2 and diag^*^” with the IPC C12Q for patents published from January 2020 for tests to identify the new coronavirus. This allows current trends in this area to be highlighted, and similarities and differences in the patented tests for SARS-CoV-1 and MERS-CoV to be compared with those related to SARS-COV-2. Table 2 shows the main patents found and the characteristics of each invention.

**Table 2:** Published patents of SARS-CoV-2 (COVID-19) tests.

**References**	**Year**	**Country**	**Product**	**Test type**	**Target**	**Sample type and/or result time**	**Benefits**
Xu et al. (94)	2020	CN	Novel nucleic acid kit for rapidly detecting SARS-CoV-2	Real-time fluorescent PCR	NS	Throat swab, nasopharynx extract, sputum. 2 h	Simple, economical, reduction of cross contamination.
Gu et al. (95)	2020	CN	Primer pair with mutation resistance	Real-time fluorescent quantitative PCR	N gene	Throat swab, alveolar lavage, saliva, blood, urine, and feces	Avoids phenomena of sensitivity reduction and false negatives
Yan et al. (96)	2020	CN	COVID-19 nucleic acid detection kit	Multiple fluorescence PCR	ORF1ab gene	Pharyngeal swab, sputum, and alveolar lavage fluid. 70 min	Good sensitivity and specificity
Wang et al. (97)	2020	CN	Novel micro-drop digital PCR kit	Digital PCR	ORF1ab and N genes	NS	Stability, repeatability, detection of low viral load, reduction of false negatives
Song and Baek (98)	2020	KR	Primer sets for detecting SARS-CoV-2	Isothermal amplification	N gene	90 min	NS
Wan et al. (99)	2020	CN	Rapid detection kit for SARS-CoV-2 dry powder LAMP	LAMP	ORF1ab and N genes	One or 2 h	Detection reagent storage at room temperature
Cui et al. (100)	2020	CN	Novel rapid detection kit for SARS-CoV-2	LAMP	ORF1ab gene	30 min	Good specificity, sensitivity, and visual identification of the result

Some of the patents identified also used PCR methods similarly to the previous patents. In one of the inventions, Xu et al. (94) published a SARS-CoV-2 rapid detection kit was developed using the fluorescent RT-PCR method and a hydrolysis probe. The kit consists of the probe, primers and a positive and negative control for detection. Three fragments of reverse transcription of segments of the virus and complementary human DNA were used as the positive and negative controls, respectively. Among the advantages of the kit described by the inventors are simplicity of use, savings on reagents and a reduction in cross contamination. The detection time was around 2 h, demonstrating good specificity by using three regions of the virus for amplification and detection. Samples obtained by pharyngeal swab, nasopharynx extract and sputum can be used in the kit [A].

Gu et al. (95) described a primer pair invention for detecting viral RNA of the new coronavirus by quantitative fluorescence PCR. The nucleocapsid gene is the target of amplification in this invention, as it allows high detection due to its low molecular weight and the generation of a high number of amplified copies. In addition, the developed primer is resistant to mutation of the virus, thereby avoiding reduced sensitivity and the generation of false negatives. The method allows the use of several samples, such as pharyngeal swab, alveolar lavage, saliva, blood, urine and feces.

Using the same PCR methodology of multiple fluorescence, Yan et al. (96) developed another kit was to simultaneously detect several SARS-CoV-2 genes. It has a 70-min detection process and can be used with pharyngeal swabs, sputum, and alveolar lavage fluids. The test showed a minimum detection limit of 2 pg/mL. Another patent by Wang et al. (97) used a digital PCR micro-drop kit to detect SARS-CoV-2 by amplifying the ORF1ab and N genes. The advantages of this test are described as being the production of results with a more direct interpretation, and greater detection sensitivity than quantitative PCR, as well as high stability, repeatability, low viral load detection capacity and a reduction in false negatives.

Other patents related to COVID-19 used isothermal amplification methods, kits and components for the detection of SARS-CoV-2 were also patented. Song Min-Seok and Baek Yoon-hee (98) developed a set of primers to allow the detection of the virus by any method of isothermal amplification within 90 min from the nucleocapsid gene. In the same vein, another invention by Wan et al. (99) presented a kit for rapid detection using the LAMP method, having as target genes ORF1ab and N. In this invention, the detection reagent was obtained by freeze drying, enabling its transport at room temperature rather than in the severe storage conditions at −20°C that these reagents often need to be kept in. The kit has a detection time of around 1–2 h and can be read by the naked eye. The LAMP method was also used in another invention by Cui et al. (100), using the ORF1ab target gene. The applied methodology makes it possible to identify the presence of the virus by a change in the color of the sample. After a detection time of 30 min, a bright green color indicates a positive result, while yellow/orange a negative result.

### Alternative Test Methodologies

Peptide-based magnetic chemiluminescence enzyme immunoassay (101, 102) is another method with good sensitivity but was not found in the search for patents. It can also be used in combination with rRT-PCR, having a rate positivity for IgG and IgM of 71.4 and 57.2%, respectively. Therefore, combining this immunoassay with real-time RT-PCR may enhance the diagnostic accuracy of COVID-19 (103–106).

Regarding the ELISA method, it is usually well-used, due to its practicality, low-cost and easy execution, ideally with each country developing with its own technologies, purifying its local antigens for good test performance (107–109).

Other methodologies not found in our survey, but with promising features are CRISPR-based methodologies (110–113), lateral flow immunoassay (5), viscoelastic testing (114), and biosensors for COVID-19 (115), being conceived as alternatives to the usual methods. In the patents identified, isothermal amplification proved to be a faster, simpler, and more sensitive method for detecting SARS-CoV-2 (116–119).

### Possible Limitations of Patented Tests

We know that antibody testing is necessary, but reagent IgG results may not guarantee new positive results for rRT-PCR, and further studies are needed to demonstrate protection against COVID-19 in reagent IgG patients (109, 120). There is a huge range of tests, but they are being used with literature-based criteria, because only mass testing can guarantee criteria for opening and closing cities (47, 121–125). The specificity and sensitivity of the tests described in this review may change due to environmental factors. According to Younes et al. (126), in ELISA assays, false-positive results may occur because protein N is the most conserved viral protein among human beta-coronaviruses. Thus, the antigens used in the kits can produce inaccurate results. Among the other possible reasons for inaccurate results are cross-linking with other coronaviruses (MERS-CoV, SARS-CoV-1) or because the antigens used have the ability to react with viruses responsible for the common cold (HKU1, 229E, OC43, NL63) in the winter when they circulate in large quantities (126). To circumvent this problem, diagnostic methods have been improved with the use of the spike protein, which detects two domains of protein S (S1 and S2) (127).

In a study by Yang et al. (128), LAMP demonstrated similar sensitivity to PCR, and specificity of 99% in the 208 clinical samples tested. This was due to the use of six to eight initiators to identify different regions. Despite being considered the gold standard, PCR is susceptible to environmental factors that can cause changes in the parameters discussed. Issues related to viral load, and slow or no antibody response can interfere in the results. Its use is recommended from the third day of symptom onset, when there is a high viral load (129, 130). Interestingly there is also a noticeable lack of technology-based products coming from developing countries and even from countries in Europe that normally play an important role in the development of diagnostic products. Thus, there is a need for greater investment to develop practical, fast and reliable technologies that can be used to provide the mass testing required to win this battle against COVID-19. Moreover, the current public patent knowledge can be a limiter in the development of new pharmaceutical product or processes, and at a particular time like a COVID-19 outbreak, so these barriers should be on the ground. Thus, these gaps in knowledge only create more doubts that build a foundation (131).

## Strengths and Limitations

Among the strengths of this study is the focus on patents in the review, which provides an overview of the situation and growth trends in a particular area of knowledge or product of interest. In addition, patents often have technological information that is not found in its entirety in articles, as companies are careful to protect their inventions, and this can provide a better overall understanding of the tests. Regarding the limitations of this review, some innovative diagnostic methods are not patented immediately, with authors preferring to have their data published quickly by means of scientific articles. Thus, most of the patents found for the diagnosis of COVID-19 are based on known methods such as PCR, isothermal amplification and ELISA. The potential bias in the identification and inclusion patent can occur because to the 18-month confidentiality period that patent offices grant to inventors. However, the search was carried out on the relevant patent databases and using comprehensive search terms and a careful selection process, in order to avoid this risk.

## Conclusion

It is known that making a fast and reliable diagnosis of a disease is of paramount importance to take fundamental measures for the control and treatment of the disease. This is particularly so in the case of viruses, especially CoVs, which affect humans in a number of different ways and can require rapid and special care in case of infection. We describe the patents that contain diagnostic methods focused on CoV, essential for the detection of SARS-CoV, MERS-CoV, and SARS-CoV-2. We also presented in this review some data from studies of trials already carried out by researchers as well as from patents aimed at other infections caused by CoV. Molecular methods, such as RT-PCR, ELISA, and isothermal amplification technologies positively contribute to simple, fast, sensitive, specific, and low-cost tests. The knowledge obtained with other types of CoV can contribute to the fight against COVID-19 and the current pandemic.

## Data Availability Statement

The raw data supporting the conclusions of this article will be made available by the authors, without undue reservation.

## Author Contributions

JN, AS, and AO analyzed and interpreted the data and performed the draft. AG, LQ-J, and LB did the critical review of intellectual content. MS realized the conception and design of the manuscript. HC and NM contributed to the final draft of the manuscript and with resources for the research. All authors contributed to the article and approved the submitted version.

## Conflict of Interest

The authors declare that the research was conducted in the absence of any commercial or financial relationships that could be construed as a potential conflict of interest.
